# Phytochemical Profiles and Biological Activities of *Frankenia* Species: A Review

**DOI:** 10.3390/molecules29050980

**Published:** 2024-02-23

**Authors:** Meyada Khaled, Rachid Ouache, Patrick Pale, Hassina Harkat

**Affiliations:** 1 Department of Pharmacy, Faculty of Medicine, Batna 2 University, Batna 05000, Algeria; meyada.khaled@univ-batna2.dz; 2 Laboratory of Physio-Toxicology, Cellular and Molecular Pathology-Biomolecules (LPTPCMB), Batna 2 University, Batna 05000, Algeria; r.ouache@univ-batna2.dz; 3 Laboratory of Organic Synthesis & Catalysis, Institute of Chemistry (UMR-CNRS 7177), University of Strasbourg, 67000 Strasbourg, France

**Keywords:** halophytes, phytochemistry, bioactivity, *Frankenia*

## Abstract

The relatively small *Frankeniaceae* family is represented by halophyte plants, growing in arid and semi-arid climates in saline, alkaline or calcareous soils. Due to their living conditions, they usually produce a large diversity of compounds, which often exhibit bioactivities. Some species of this genus have long been used as traditional herbal medicines to treat dysentery, diarrhea, gonorrhea, vaginal leucorrhea, respiratory diseases and wounds. To date, several studies on either phytochemical or pharmacological aspects, or both, have revealed that this genus is a rich source of diverse and novel bioactive chemicals, including phenolics, flavonoids, alkaloids and fatty acids. This review describes all the reported chemical profiles of *Frankenia* species, as well as the corresponding biological properties, when available. The aim of this review is to show the potential of these plants for various applications, especially therapeutic ones.

## 1. Introduction

According to a very recent report from the World Health Organization (WHO), ‘traditional medicine has a long history of contributing to conventional medicine and continues to hold promise’ [1]. Indeed, since early times, human beings have learned how to address their health problems and various so-called traditional medicines have emerged all over the world and are still used, according to the 2019 WHO global report on traditional and complementary medicine [2]. These traditional medicines usually rely on natural products and mixtures of them, issued mostly from plants [3], but also from animals and microorganisms [4]. 

**Figure 1:**
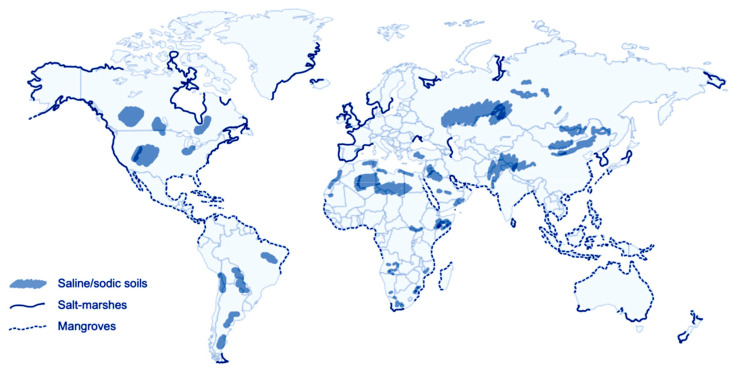
World distribution of halophytes (adapted with permission from [7]).

Marine environments are known for their high biodiversity. Among them, coastal environments exhibit specific plants able to grow in highly saline areas, often under severe variations in temperature, light intensity and drought. These plants, named halophytes, are not limited to such coastal areas but can be found in a diverse array of highly saline soils (Figure 1) [5]. To withstand such severe conditions, these plants have developed several ways to control and/or take away salt, but they also exhibit strong antioxidant systems composed of enzymes and highly bioactive secondary metabolites, such as phenolic compounds and alkaloids [6]. Probably for these reasons, halophytes are traditionally used in folk medicine for their curative properties against infectious diseases [7]. Hence, halophytes are currently gaining interest due to their nutraceutical potential, powerful antioxidant abilities and therapeutic significance in treating a variety of pathologies [8].

Among the halophytes, the *Frankeniaceae* family constitutes a relatively small family with originally 2 to 4–5 genera, but a recent taxonomic revision based on molecular phylogenetic studies retained the *Frankeniaceae* as a single genus, *Frankenia* [9]. The latter contains between 70 and 82 species that are found in deserts and sandy coastal locations with dry areas [9]. Similarly, the *Frankeniaceae* and *Tamaricaceae* families were considered as a pair of families that together made up the order Tamaricales, however, genetic studies have allowed them to be distinguished [9,10].

**Figure 2:**
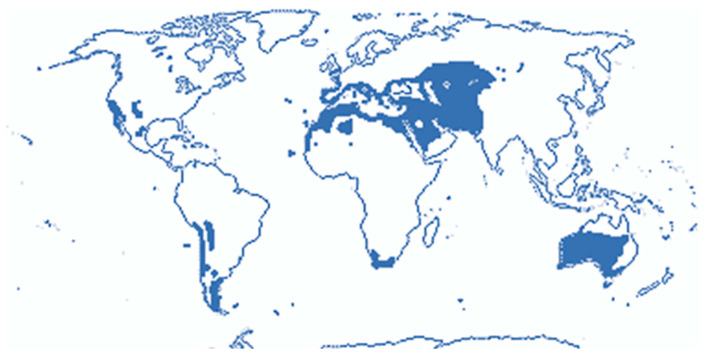
World distribution of *Frankenia* species (adapted from [11]).

The shrubby and herbaceous species of *Frankenia* are known to mainly grow in arid and semi-arid climates in extremely saline, alkaline or calcareous soils. They can be found on all continents but are most common in the Western Hemisphere, particularly in the Mediterranean region up to the Middle East (Figure 2) [11]. Indeed, *Frankenia* species have been recorded in North Africa, especially in Algeria and Tunisia, as well as in Egypt, Portugal, Spain and France, but also in Turkey, Syria, Lebanon, Jordan and Palestine. They can also be found in Iraq and neighboring regions, such as Qatar, Kuwait and Iran (Table 1).

Despite their relevance in medicine and industry, studies about the *Frankenia* genus are unexpectedly limited, probably due to the scarcity of these plant species. Nonetheless, only a few species have been investigated in some detail (Table 1). Their chemical profile and/or their biological properties have been explored, revealing a wide variety of natural products and bioactivities.

The purpose of this study was to collect and systematically review the published phytochemical compositions and biological activities of the medicinal *Frankenia* species.

## 2. Phytochemical Profile of *Frankenia* Plants

From the studies mentioned above, more than 200 phytocompounds obtained from *Frankenia* extracts have already been identified. Among them, polyphenols, such as phenolic acids and flavonoids, are the major constituents and essential chemotaxonomic indicators. Further isolated compounds include alkaloids, terpenoids, steroids, fatty acids and other molecules.

### 2.1. Polyphenols

#### 2.1.1. Phenolics

Phenolics are a broad category of chemical compounds that have one or more hydroxyl groups linked to at least one aromatic hydrocarbon ring [37]. Due to their hydroxylated conjugated structure, phenolic compounds have considerable potential as antioxidants [38]. Furthermore, their stability could allow their use as therapeutic agents.

**Figure 3:**
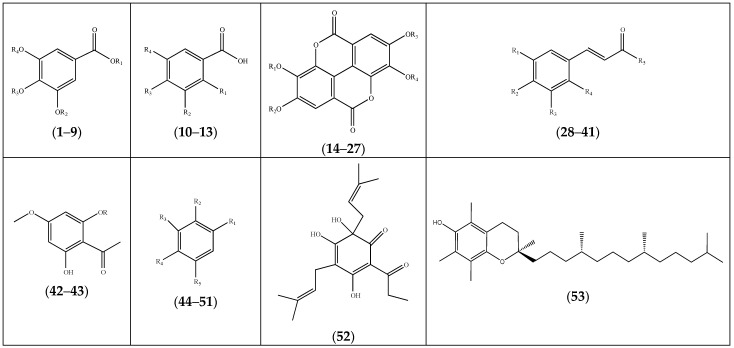
Chemical skeleton of phenolics (**1**–**53**) isolated from the genus *Frankenia* (for detailed structures, see Table 2).

Phenolic natural products are abundant in the *Frankenia* genus, especially in *F. laevis*, and exhibit a large variability (Figure 3). They were mainly represented by gallic acid (**1**–**9**), hydroxybenzoic acid (**10**–**13**), ellagitannins (**14**–**27**) and hydroxycinnamic acid (**28**–**41**) derivatives (Table 2). Other phenolic compounds (**42**–**53**) were also identified (Figure 3). The dihydroxybenzenes (**44**) and (**45**) were present in significant amounts in *F. pulverulenta* [39]. Additionally, compounds (**10**), (**28**) [23] and (**31**) [39] were the most representative compounds in *F. thymifolia*, followed by (**29**) [23].

#### 2.1.2. Flavonoids

**Figure 4:**
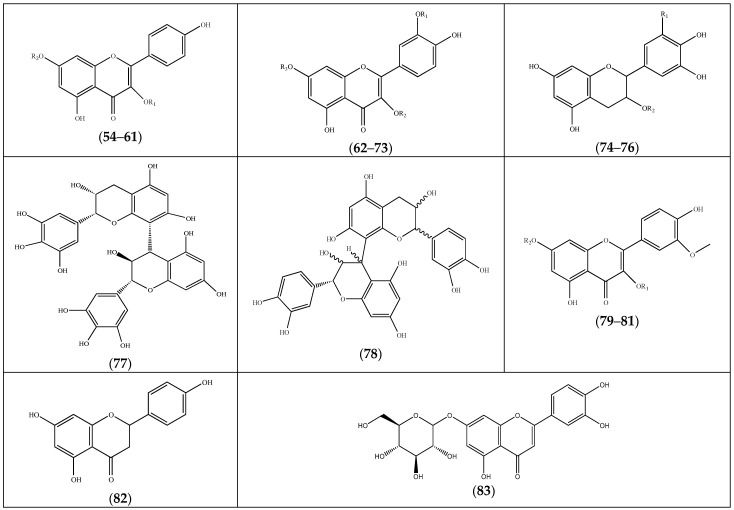
Chemical structures of flavonoids (**54**–**83**) isolated from the genus *Frankenia* (for more detailed structures, see Table 3).

The *Frankenia* species, especially *F. laevis*, *F. pulverulenta* and *F. thymifolia*, contain a wide range of flavonoids (Table 3), mainly represented by flavonols, such as kaempferol (**54**–**61**), quercetin (**62**–**73**), catechin (**74**–**78**) and isorhamnetin (**79**–**81**) derivatives. The compounds (**74**) and (**75**) were the most representative flavonoids in *F. thymifolia* and *F. pulverulenta*, respectively [23,39]. Additionally, the flavanone (**82**) and the *O*-glycosylated flavone (**83**) were described in *F. thymifolia* (Figure 4).

#### 2.1.3. Lignans

**Figure 5:**
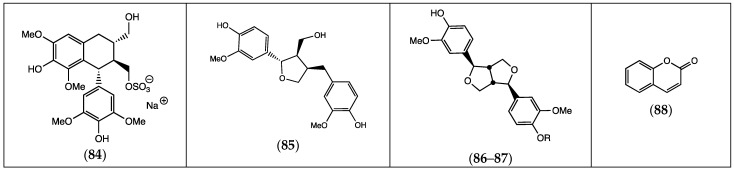
Chemical structures of lignans (**84**–**87**) and coumarins (**88**) isolated from the genus *Frankenia* (for the detailed structures of **86–87**, see Table 4).

Lignans do not appear to be extremely prevalent in *Frankenia* (Table 4). The arylated tetralin derivative (**84**) is the most common lignan found in these plants. A few tetrahydrofuranic lignans (**85**–**87**) have also been isolated and identified (Figure 5).

#### 2.1.4. Coumarins

So far, a single example of coumarin, the simplest one (**88**), has been isolated and characterized from a *Frankenia* species (Table 4 and Figure 5).

### 2.2. Alkaloids

**Figure 6:**
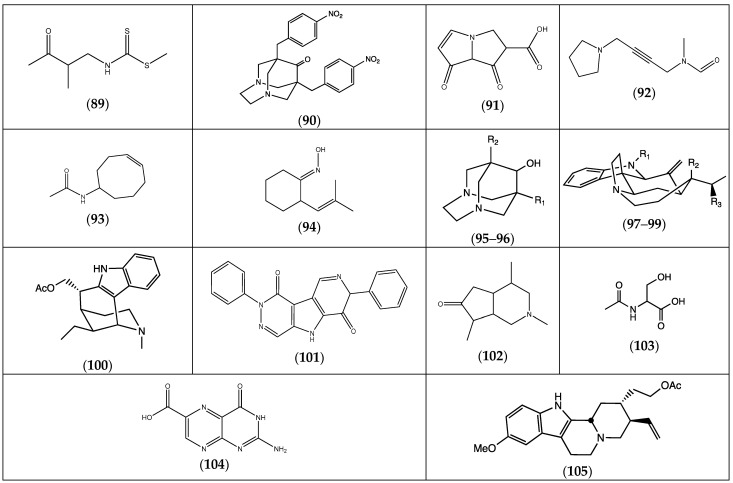
Chemical structures of alkaloids (**89**–**105**) isolated from the genus *Frankenia* (for more detailed structures, see Table 5).

Surprisingly, only a few alkaloids could be found in *Frankenia* species, and they were mainly detected in *F. pulverulenta* (Table 5). The phytochemical investigation of this species has led to the identification of several compounds with a wide variety of structures (**89**–**101**). The alkaloid dihydrotecomanine (**102**) was detected in both *F. pulverulenta* and *F. hirsuta* (Figure 6). In *F. aucheri(hirsuta)*, an α-amino acid metabolite, the *N*-acetyl serine (**103**), could also be observed and characterized. The same species also contains another peculiar amino acid with a heterocyclic core, the pterin-6-carboxylic acid (**104**), but an indoloquinolizine derivative (**105**) was also identified.

### 2.3. Terpenoids

**Figure 7:**
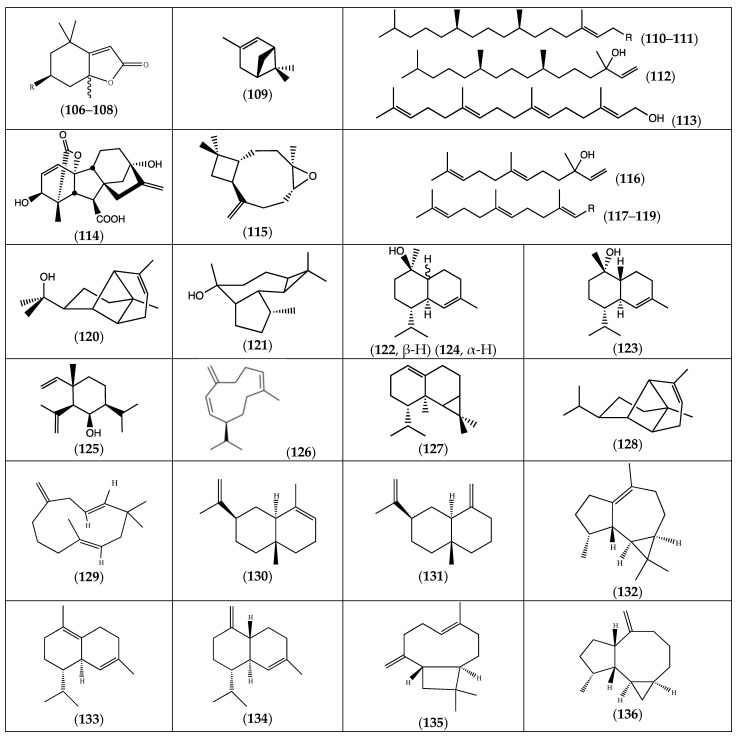
Chemical structures of terpenoids (**106**–**136**) isolated from the genus *Frankenia* (for more detailed structures, see Table 6).

As important building blocks in biosynthesis, terpenoids are widely represented in living species. This large class of natural compounds is extremely prevalent in the plant kingdom, where they play key roles in plant defense and communication [44]. They are frequently found in plant essential oils. Therefore, it is natural to find them in plants of the *Frankenia* genus. However, in *Frankenia* species, most of them have been identified as sesquiterpenes. Nevertheless, some mono- and diterpenes have also been found (Figure 7).

#### 2.3.1. Monoterpenes

Monoterpenes are not common among *Frankenia* species. It was only in 2021 and 2022 that a few monoterpenes were identified in, respectively, *F. pulverulenta* and *laevis*. Indeed, the phytochemical analysis of *F. laevis* extracts revealed the presence of three tetrahydrobenzofuran-2(4*H*)-ones (**106**–**108**). In *F. pulverulenta* extracts, the bicyclic α-pinene (**109**) was detected (Table 6).

#### 2.3.2. Diterpenes

A few compounds have been reported as diterpenes from *Frankenia* species. The acyclic diterpenoids (**110**–**112**) were isolated from *F. laevis*, while (**113**) and its derivative gibberellic acid (**114**) were present in the essential oil (EO) and methanolic leaf extract of *F. pulverulenta*, respectively (Table 6).

#### 2.3.3. Sesquiterpenes

Sesquiterpenes are abundant in the *Frankenia* genus. Overall, 22 compounds belonging to this subclass of terpenoids were identified in the EO of *F. laevis* and *F. pulverulenta*, including eleven oxygenated sesquiterpenes (**115**–**125**) and eleven sesquiterpene hydrocarbons (**126**–**136**) (Table 6).

Nerolidol (**116**) and farnesyl acetate (**119**) were the most widespread sesquiterpenes present in *F. laevis* [43]. Furthermore, β-caryophyllene (**135**) was the major compound detected in *F. pulverulenta*. The second major compounds were cadinene (**134**), allo-aromadendrene (**136**), copaene (**128**) and ledol (**121**) [18].

However, the later terpenes (**134**), (**135**) and (**136**) were found to be present at a much lower amount in *F. laevis* [43].

### 2.4. Steroids

**Figure 8:**
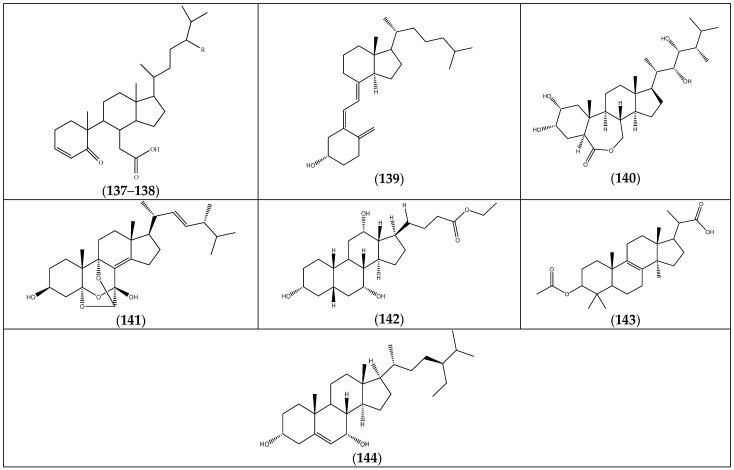
Chemical structures of steroids (**137**–**144**) isolated from the genus *Frankenia* (for more detailed structures of **137**–**138**, see Table 7).

Although they do not seem to be common in *Frankenia* species, steroids have nevertheless been isolated and characterized (Figure 8 and Table 7). The majority of them have been isolated from *F. foliosa* and identified as secosteroids (**137**–**141**) [35]. It is worth noticing that the latter included vitamin D (**139**) as well as the unusual eringiacetal A (**141**). In addition, the two steroids (**142**) and (**143**) have been identified in *F. pulverulenta* [24], while the steroid (**144**) has been detected in *F. hirsuta* [36].

### 2.5. Alkanes and Alkenes

Long-chain alkanes are common in terrestrial plants, especially as part of their cuticular leaf wax. Therefore, alkanes are quite common in *Frankenia* species (Table 8). Overall, 15 alkane chemicals (**145**–**158**) were reported in both *F. laevis* and *F. pulverulenta* [18,43]. 

Most of these alkanes exhibit linear and long carbon chains, containing from 17 to 35 carbons. So far, a single example of an α-methylated chain alkane (**156**) has been reported in *F. laevis*. Similarly, the C20 linear alkane eicosane (**159**) has so far only been characterized in *F. hirsuta* [36].

In contrast, long-chain alkenes seem to be quite rare in *Frankenia* species. Indeed, only two alkenes (**160**) and (**161**) have been reported as the only alkenes in the genus [36,43]. Both exhibit a terminal vinyl group within a linear chain (C19 and C22, respectively).

### 2.6. Fatty Acids and Esters

Fatty acids and esters are ubiquitous in all living organisms and are essential to them, as they serve as membrane constituents, modulators in glycerolipids and as carbon and energy reserves in triacylglycerols, but also as signal molecules [45].

The *Frankenia* plants contain various fatty acids and esters. At least 20 different fatty acids and fatty acid esters were found within members of the genus *Frankenia* (Table 9), and grouped as saturated (**162–171**), monounsaturated (**172**–**173**) and polyunsaturated (**174**–**178**) fatty acids, saturated fatty acid methyl esters (**179**–**180**) and unsaturated fatty acid methyl esters (**181**–**183**). Palmitic acid (**167**) was the major compound of *F. laevis*, followed by (**181**) [43]. In addition, (**167**), (**173**) and (**174**) were reported as the major fatty acids in the oil of *F. thymifolia* [23].

### 2.7. Other Compounds

**Figure 9:**
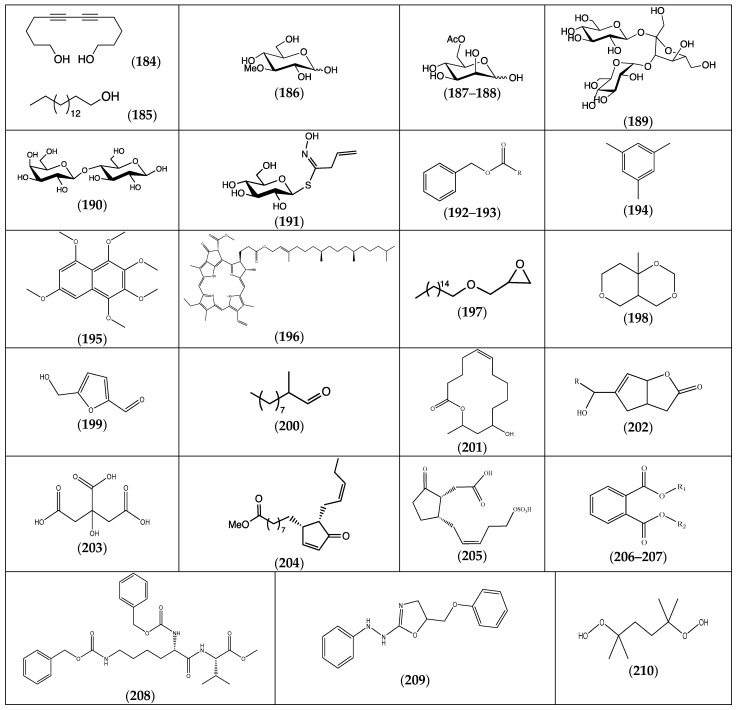
The chemical structures of various compounds (**184**–**210**) isolated from the genus *Frankenia* (for more detailed structures of **206**–**207**, see Table 10).

In addition to the large well-known natural product families mentioned above, compounds from other classes of natural chemicals have also been detected (Figure 9 and Table 10). A large variety of compounds was identified, such as alcohols (**184**–**185**), glycosides (**186**–**191**), aromatic compounds (**192**–**195**), heterocyclic compounds (**196**–**198**), aldehydes (**199**–**200**), ketones (**201**–**202**), organic acids (**203**–**205**) and esters (**206**–**208**). 

Long-chain alkyl alcohols, unsaturated or not, (**184**–**185**) were detected in *F. pulverulenta* and *F. laevis*, respectively [24,43]. Surprisingly, the same hexadecane-1-ol (**185**) found in *F. laevis* was also observed in *F. hirsuta* but as its glycidyl ether (**197**) [32].

Highly abundant in organisms, especially in plants, glycosides were only scarcely found in *Frankenia* species. The common monosaccharides, glucose and mannose, were both detected in *F. pulverulenta* and *F. hirsuta*, but as, respectively, their 3-*O*-methylated or 6-*O*-acetylated derivatives (**186**–**188**) [24,32]. A desulfonylated allyl glucosinolate was also detected in *F. hirsuta*, (**191**) [32]. Such a sinigrin derivative is usually found in the *Brassicaceae* family. A di- and a trisaccharide were also detected in *F. pulverulenta*. The disaccharide was unexpectedly characterized as lactose (**190**) [24], while the trisaccharide was assigned as a β-analog of melezitose (**189**) [24]. Interestingly, the aromatized form of glucose, i.e., hydroxymethylfurfural (HMF) (**199**), was also detected in *F. hirsuta* [32].

Among the other aromatic compounds found (**192**–**195**), a demetallated chlorophyll, i.e., pheophytin A, was observed in *Frankenia* species, and more precisely in *F. laevis* [13]. Alternatively, the *F. hirsuta* species seems relatively rich in aromatic compounds, since the simple 1,3,5-trimethylbenzene (**194**), phthalate esters (**206**–**207**) and various benzyl or phenyl derivatives (**192**–**193** and **208**–**209**) have been observed [32,36]. A hexamethoxylated naphthalene derivative, i.e., (**195**), was detected in *F. thymifolia* [22].

A few jasmonoids, i.e., (**204**–**205**), were found in *F. laevis* [13], as well as a related cyclopentenyl bicyclic lactone in *F. pulverulenta* [24]. Interestingly, the macrolactone (**201**)**,** related to the methyl ester (**183**)**,** was also detected in *F. pulverulenta* [24].

Furthermore, the hydrazine (**209**) and the stable peroxide (**210**) were both reported from the *F. hirsuta* species [32].

### 2.8. Phytochemical Outcome

**Figure 10:**
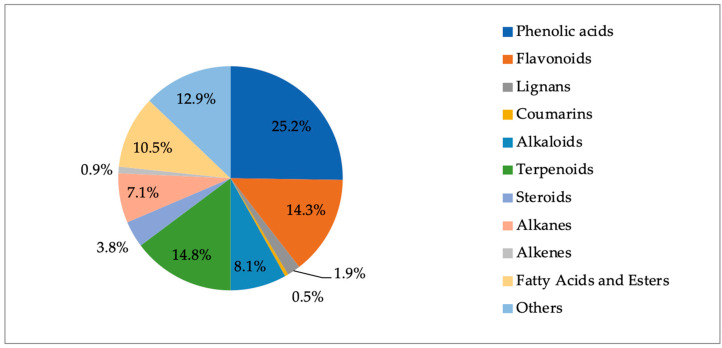
Repartition of the various phytochemicals so far observed in and/or isolated from *Frankenia* plants.

The phytochemical compositions of the various *Frankenia* species collected above reveal the rich chemical content of these plants and the variety of chemicals that have been detected, or isolated and characterized. These plants mainly produce phenol derivatives, which represent around one-quarter of all the so far identified chemicals. The other chemicals mostly observed belong to the flavonoid and terpenoid families (14–15% each), while alkaloids, fatty acids and esters represent approximately 10% of all *Frankenia* phytochemicals (Figure 10).

**Figure 11:**
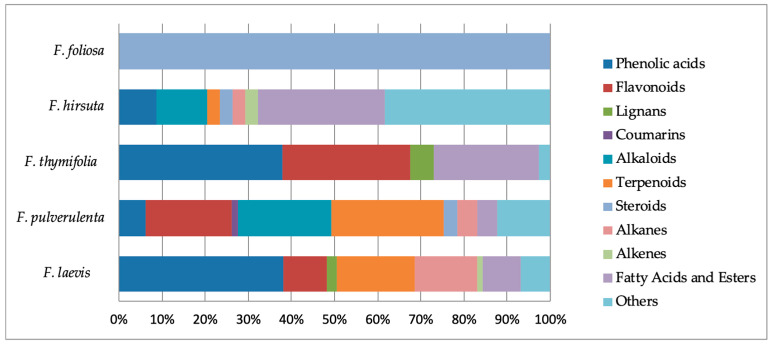
Distribution of phytochemicals in typical *Frankenia* species.

Overall, *Frankenia* species might be regarded as potentially rich sources of phenolics, flavonoids, sesquiterpenes and fatty acids or esters. Nevertheless, the phytochemical repartition is quite different from one species to another, as revealed in Figure 11.

Indeed, *F. laevis* and *F. thymifolia* are particularly rich in phenol derivatives, while *F. pulverulenta* and *F. hirsuta* exhibit less than 10% of such chemicals. Similarly, fatty acids or esters are mostly present in *F. hirsuta* and *F. thymifolia*, while they represent less than 10% of the phytochemical content of *F. pulverulenta* and *F. laevis*.

Flavonoids are mostly present in *F. thymifolia* and *F. pulverulenta,* and to a lesser extent in *F. laevis*. However, they are surprisingly almost absent in other species. The same surprising repartition can be observed for terpenoids, which are mostly present in *F. pulverulenta* and *F. laevis*, but also to a small extent in *F. hirsuta* and almost absent in other species.

Among *Frankenia* species, *F. hirsuta* seems to present the largest diversity. Indeed, in addition to the large and ubiquitous classes of compounds mentioned above, *F. hirsuta* also contains alkaloids, steroids, (oligo)saccharides and aromatic derivatives, as well as unexpected hydrazine and peroxide derivatives.

Such different repartitions may be useful as chemotaxonomic tools, complementing others. Indeed, previous research demonstrated that plants of the genus *Frankenia* may produce sulfated chemicals, where they serve as an indirect chemotaxonomic marker. Furthermore, their presence has been correlated to their affinity for saline environments [22].

## 3. Biological Activities of *Frankenia* Plants

### 3.1. In Traditional Medicine

As was reminded in the introduction, traditional medicine has a long history in human health and various variants have been developed around the world and are still practiced nowadays. In countries with limited access to modern therapy, traditional medicine is frequently the major source of primary healthcare requirements [1,2,46].

Used as traditional medicinal plants, *Frankenia* species appear to play a prominent role in the treatment of various diseases. Due to their astringent properties, *Frankenia* species are utilized in Asian and African (especially in Morocco) folk medicine for gargling or for topical application, either as tinctures or as herbal tea, e.g., with *F. laevis* or *F. thymifolia*. They are also used in these countries to treat a variety of clinical disorders, such as dysentery, diarrhea, gonorrhea, vaginal leucorrhea, mucus releases from the nose and catarrh-induced infections, again as plant infusions, with, e.g., *F. pulverulenta*, or as stupe, depending on the localization [17,18,47].

Gargle and decoction generated from the entire plant of *F. pulverulenta* are widely used in local medicine by the inhabitants of the Onaizah province in Saudi Arabia and are mostly used orally for their analgesic and carminative properties [18]. Also in Saudi Arabia, the powdered rhizome of *F. aucheri (hirsuta)* combined with milk is used to stimulate lactation in cows and camels, particularly in the winter [48].

It has also been reported that Puna inhabitants in South America used *F. triandra* as a forage but also as antiseptic in folk medicine [34].

Additionally, some *Frankenia* species can be converted into sticky glue mixtures, due to their specific natural product contents, e.g., kaempferol, quercetin and tannin. Therefore, they are used in totally different applications, notably to stick blade cutting edges and to seal stoneware (e.g., *F. hirsuta*) [32].

Overall, *Frankenia* plant species may thus be viewed as promising prospects for different applications in industry, and mostly in pharmaceutical applications.

### 3.2. In Vitro Biological Activities

Secondary plant metabolites, which are produced in large amounts by plant species, are crucial components for supporting human health. They contribute to the medicinal properties of plant species as antioxidants, anti-inflammatory, anti-carcinogenic and antibacterial agents [3,4], along with other capacities [46].

Because the large diversity of natural compounds discovered in *Frankenia* species mostly belong to well-known families of bioactive compounds, it is expected that these plants exhibit the corresponding biological activities. Therefore, several works have been performed to check these bioactivities. They are listed below.

#### 3.2.1. Antioxidant Activity

The antioxidant activity of *Frankenia* species has been assessed using several methods, including the 2,2-diphenyl-1-picrylhydrazyl (DPPH) free-radical-scavenging analysis, 2,20-azino-bis(3-ethylbenzthiazoline-6-sulfonic acid) (ABTS) cation radical trapping, ferric ion reducing antioxidant power (FRAP), metal chelating activity (MCA), including copper (CCA)- and iron (ICA)-chelating activities, oxygen radical absorbance capacity (ORAC) assay and β-carotene oxidation test. The corresponding details have been collected in Table 11.

The aqueous acetone [17], methanol [13] and ethanol [41] extracts of *F. laevis* exhibited spectacular in vitro radical scavenging and copper chelating properties. However, the dichloromethane extract from this species was only able to chelate iron, probably due to the presence of various phenolics and flavonoids that can act as phytochelators [49], such as gallic acid, kaempferol and quercetin derivatives (see Table 2 and Table 3 and Figure 3 and Figure 4).

Likewise, *F. pulverulenta* ethyl acetate [19] and methanolic [39] extracts were investigated using DPPH, ABTS and ORAC assays, and potent antioxidant activity was reported. The antioxidant activity of the aqueous acetone extract of *F. pulverulenta* was also assessed [17]. The antioxidant potency can be linked to the well-known antioxidant compounds gallic acid (**1**), *p*-coumaric acid (**34**), quercetin (**62**) and catechin (**74**) which are abundantly present in this species. Ben Mansour et al. [21] demonstrated, in 2016, that *F. thymifolia* ethyl acetate extracts exhibited strong antioxidant activity in both shoots and roots. Furthermore, this extract has the highest TPC and antioxidant capacities [42]. Other studies investigated the methanolic and chloroformic extracts of *F. thymifolia* and demonstrated that the methanolic extract exhibited better antioxidant activity, again linked to the high level of phenolic compounds, including salicylic acid (**10**), cinnamic acid (**28**), 2-hydroxycinnamic acid (**29**), chlorogenic acid (**31**) and catechin (74) [23,39]. Torres Carro et al. showed, in 2016, the significant antioxidant activity of the ethanolic and soxhlet of *F. triandra* evaluated by the β-carotene assay [34].

Overall, plants from the *Frankenia* family are rich in polyphenols, and this richness is often, if not always, correlated to the strong antioxidant properties these plants exhibit [13,17].

**Table 11:** Collected experimental data on the antioxidant activity of *Frankenia* species.

*Frankenia* Species	Extract/ Fraction	Organ	Assay								Ref.
			DPPH	ABTS	FRAP	CCA	ICA	ORAC	β-Carotene	MCA	
*F. laevis*	aqueous acetone	AP	IC_50_ = 0.12 mg/mL	IC_50_ = 0.18 mg/mL	n.d	IC_50_ = 0.44 mg/mL	IC_50_ > 1 mg/mL	n.d	n.d	n.d	[17]
	methanol	AP	EC_50_ = 0.25 mg/mL	EC_50_ = 0.65 mg/mL	EC_50_ = 0.51 mg/mL	EC_50_ = 0.78 mg/mL	EC_50_ > 1 mg/mL	n.d	n.d	n.d	[13]
	dichloromethane		EC_50_ > 1 mg/mL	EC_50_ > 1 mg/mL	EC_50_> 1 mg/mL	EC_50_ > 1 mg/mL	EC_50_ = 0.76 mg/mL	n.d	n.d	n.d	
	ethanol	Sh	IC_50_ = 48.3 µg/mL	IC_50_ = 93.4 µg/mL	n.d	n.d	IC_50_ = 240 µg/mL	n.d	n.d	n.d	[41]
*F. pulverulenta*	aqueous acetone	AP	IC_50_ = 0.10 mg/mL	IC_50_ = 0.15 mg/mL	n.d	IC_50_ = 0.30 mg/mL	IC_50_ = 0.50 mg/mL	n.d	n.d	n.d	[17]
	ethyl acetate	Sh	586 mg TE/g E	1453 mg TE/g E	n.d	n.d	n.d	821 mg TE/g E	n.d	37 mg EDTA/g E	[19]
		R	750 mg TE/g E	1319 mg TE/g E	n.d	n.d	n.d	1054 mg TE/g E	n.d	23 mg EDTA/g E	
	methanol	AP	1090.4 mg TE/g E	3621.43 mg TE/g E	n.d	n.d	n.d	58.08 mg TE/g E	n.d	71.98 mg EDTA/g E	[39]
*F. thymifolia*	methanol	AP	IC_50_ = 99 µg/mL	n.d	n.d	n.d	EC_50_ = 120 µg/mL	n.d	IC_50_ = 11 µg/mL	n.d	[23]
	chloroform		IC_50_ = 120 µg/mL	n.d	n.d	n.d	EC_50_ = >1000 µg/mL	n.d	IC_50_ = >1000 µg/mL	n.d	
	ethyl acetate	AP	n.d	n.d	n.d	n.d	n.d	n.d	71.66 ± 1.24% at 100 mg/mL	n.d	[21]
	*n*-butanol		n.d	n.d	n.d	n.d	n.d	n.d	50.83 ± 1.65% at 100 mg/mL	n.d	
	chloroform		n.d	n.d	n.d	n.d	n.d	n.d	49.91 ± 1.06% at 100 mg/mL	n.d	
*F. triandra*	ethanol	AP	n.d	SC_50_ = 37.22 µg/mL	n.d	n.d	n.d	n.d	IC_50_ = 41.24 µg/mL	RC_50_ = 15.08 µg/mL	[34]
	soxhlet		n.d	SC_50_ = 35.99 µg/mL	n.d	n.d	n.d	n.d	IC_50_ = 43.33 µg/mL	RC_50_ = 16.53 µg/mL	

AP: aerial parts, Sh: shoots, R: roots. DPPH: 2,2-diphenyl-1-picrylhydrazyl. ABTS: 2,20-azino-bis(3-ethylbenzthiazoline-6-sulfonic acid. FRAP: ferric ion reducing antioxidant power. MCA: metal chelating activity. CCA: copper chelating activity. ICA: iron chelating activity. ORAC: oxygen radical absorbance capacity. n.d: not determined. EC_50_: half maximal effective concentration. IC_50_: half maximal inhibitory concentration. SC_50_: scavenging concentration 50%. SC_50_: concentration for 50% reduction. Ref.: references.

#### 3.2.2. Antimicrobial Activity

Bacteria were involved in many of the most devastating diseases and massive epidemics in human history, before the discovery of antibiotics. Due to the misuse of the latter, bacteria have now developed resistance to the commonly used antibiotics [50]. Therefore, it is imperative to identify new and advanced chemical agents in order to have more productive resistance to microorganisms [50]. Since synthetic chemicals are related to adverse effects and harmful residues, novel antibacterial, antifungal, antiviral and antiparasitic drugs from plant sources must be developed worldwide [31,51,52]. Accordingly, a few *Frankenia* species were collected and screened for their antimicrobial activities. The corresponding details have been collected in Table 12.

Jdey et al. showed, in 2017, that the ethanolic extract of *F. laevis* significantly inhibited the development of both Gram-positive (Gram+) and Gram-negative (Gram−) bacteria engaged in their study [41]. All these strains were indeed inhibited by more than 55%, and the best inhibitions were observed for *Micrococcus luteus* (83%) and *Salmonella enterica* (77%) at a concentration of 1 mg/mL. This antibacterial effect may be attributed to the chlorogenic acid (**31**) and catechin (**74**) contained in this species, probably by inducing structural or functional damage to the bacterial cell membranes [53]. Similarly, Saïdana et al. [43] demonstrated, in 2010, that EO from the aerial parts of *F. laevis* was efficient against *Staphylococcus aureus, Staphylococcus epidermidis, Micrococcus luteus, Escherichia coli* and *Salmonella typhimurium*. According to the authors, the antimicrobial activity of the EO can be attributed to the presence of fatty acids [54] such as palmitic acid (**167**), fatty acid esters like methyl linoleate (**181**), sesquiterpenes [55] such as farnesyl acetate (**119**)**,** aromatic compounds [56] such as benzyl benzoate (**192**) and benzyl cinnamate (**193**), and, to a lesser extent, eugenol (**49**), β-caryophyllene (**135**), phytol (**110**), isophytol (**112**), (*E*, *E*)-farnesol (**117**) and hexadecanol (**185**). However, no significant effect on *Pseudomonas aeruginosa* was detected [43]. This Gram− bacteria has already been demonstrated to be less susceptible to the action of several other plant EOs [57]. The antifungal activity of the EO was also investigated. Despite the presence of eugenol (**49**) and β-caryophyllene (**135**) in the oil composition, known to have antifungal effects [58], none of the tested fungi were successfully inhibited by the EO at the tested doses. This may be explained by the low amounts of such chemicals in the EO [57].

The antibacterial and antifungal activity of EO from *F. pulverulenta* was also investigated [18]. Despite being rich in β-caryophyllene (**135**), which represents its main constituent (32%), this EO did not prevent bacterial growth. This finding appears contradictory to previous research, which showed that the presence of β-caryophyllene enhances the biological activities of EO, including their antibacterial activity [59,60]. Furthermore, the *F. pulverulenta* EO displayed poor antifungal activity and was exclusively efficient against the basidiomycete *Rhizoctonia solani* [18]. In 2011, Megdiche-Ksouri et al. investigated the activity of methanolic (polar) and chloroformic (less polar) extracts from *F. thymifolia* against five bacteria and one fungus [23]. The chloroformic extract provided the best performance, being active against all the evaluated bacterial strains. Similar inhibition results have been observed with other halophytes (e.g., sea holly, sea fennel) [61]. Such an outcome has been correlated to the polarity of the extracting solvent and could be attributed to the presence of lipophilic compounds in these extracts. Indeed, it has been demonstrated that long-chain unsaturated fatty acids, notably oleic (**172**) and linoleic (**174**), exhibit a strong inhibiting activity against mycobacteria [62]. Furthermore, it has been reported that the relatively lipophilic flavonoids catechin (**74**) and epigallocatechino-3-gallate (**76**) exhibit protective and antibacterial effects [63]. Likewise, the *n*-butanol fraction from *F. thymifolia* exhibited a stronger antibacterial effect against all tested bacterial strains compared to the ethyl acetate fraction (*Pseudomonas aeruginosa* was the most vulnerable strain) [42]. The extracts investigated also presented anti-leishmanial and antiamoebic effects against *Leishmania amazonensis* and *Acanthamoeba castellanii*, respectively. The antiparasitic capacities of these extracts may be related to the presence of quercetin (**62**) [64].

Canli et al. demonstrated the antibacterial and antifungal activity of *F. hirsuta* ethanolic extract against seventeen bacteria and one fungus [36]. Except for the Gram˗ bacteria *Enterobacter aerogenes* and *Escherichia coli*, all of the examined strains were sensitive to the antimicrobial action of the *F. hirsuta* extract. The most sensitive strains were Gram+ bacteria, especially *Staphylococcus epidermidis* and *Enterecoccus faecium*, compared to Gram˗ bacteria. Such antibiotic activity was again associated with the presence in this extract of oleic and linoleic acid in high amounts [65]. The concomitant presence of mesitylene (**194**)**,** eudesmic acid (**8**) and stearic acid (**169**) also suggested a possible role in the *F. hirsuta* antibacterial activity, because some mesitylene derivatives [66], eudesmic acid [67] and stearic acid analogs [68] are known as antibacterial agents.

The difference in sensitivity to plant extracts between Gram+ and Gram− bacteria observed for *F. hirsuta* ethanolic extract could be generalized according to Canli et al. in other studies [69].

It is worth reminding here that the antibacterial properties of certain unsaturated fatty acids (oleic (**172**) and linoleic (**174**) acids) and, to some extent, of palmitic and stearic acids (**167**, **169**) are linked to their ability to inhibit enoyl-acyl carrier protein reductase (FabI) activity [65,70].

The antibacterial activity of an ethanolic extract of *F. triandra* was also investigated [71]. The antischistosomal action of the methanol extract from *F. hirsuta* can also be found [31]. Interestingly, the acetonic and methanolic extracts derived from the aerial part of *F. pulverulenta* exhibited antiviral activity against *Herpes simplex* virus type 1 (HSV-1) at a dose of 500 μg/mL [72]. *F. pulverulenta* is known to contain flavonoids, including the 7-bisulfate-3-glucuronide of kaempferol (**61**), isorhamnetins (**79**–**80**) and quercetin (**62**) [27,72]. As some flavonoids, notably quercetin and, to a lesser extent, catechin and hesperetin, have been reported to possess antiviral capacities against a number of viruses, including HSV-1, the antiviral activity of *F. pulverulenta* extracts may be linked to its content of flavonoids [73].

These investigations and their results clearly suggest that *Frankenia* plants might be a valuable source of antimicrobial substances.

**Table 12:** Collected experimental data on the antimicrobial activity of *Frankenia* species.

Microbial Strain	*Frankenia* Species	Extract/ Fraction/EO	Organ	Assay	MIC (mg/mL)	MSI (%)	IZ (mm)	Ref.
**Gram+ bacteria**								
*Micrococcus* *luteus*	*F. laevis*	ethanol	AP	microdilution	n.d	83.16 ± 0.38	n.d	[41]
	*F. laevis*	EO	AP	disc diffusion	0.5	n.d	n.d	[43]
*Staphylococcus aureus*	*F. laevis*	ethanol	AP	microdilution	n.d	66.66 ± 1.25	n.d	[41]
	*F. laevis*	EO	AP	disc diffusion	0.5	n.d	n.d	[43]
	*F. pulverulenta*	EO	AP	well diffusion	n.d	n.d	-	[18]
	*F. thymifolia*	methanol chloroform	Sh Sh	disc diffusion disc diffusion	n.d n.d	n.d n.d	8.6 8.0	[23]
	*F. thymifolia*	*n*-butanol ethyl acetate	AP	disc diffusion disc diffusion	n.d n.d	n.d n.d	9.0 11.0	[42]
*Staphylococcus epidermidis*	*F. laevis*	EO	AP	disc diffusion	0.8	n.d	n.d	[43]
	*F. hirsuta*	ethanol	H	disc diffusion	n.d	n.d	16.0	[36]
*Enterococcus* *faecium*	*F. thymifolia*	methanol chloroform	Sh Sh	disc diffusion disc diffusion	n.d n.d	n.d n.d	9.5 8.5	[23]
	*F. hirsuta*	ethanol	H	disc diffusion	n.d	n.d	16.0	[36]
**Gram− bacteria**								
*Escherichia coli*	*F. laevis*	ethanol	AP	microdilution	n.d	56.18 ± 1.13	n.d	[41]
	*F. pulverulenta*	EO	AP	well diffusion	n.d	n.d	-	[18]
	*F. laevis*	EO	AP	disc diffusion	0.8	n.d	n.d	[43]
	*F. thymifolia*	methanol chloroform	Sh Sh	disc diffusion disc diffusion	n.d n.d	n.d n.d	6.0 10.0	[23]
	*F. thymifolia*	*n*-butanol ethyl acetate	AP	disc diffusion disc diffusion	n.d n.d	n.d n.d	8.0 7.0	[42]
	*F. hirsuta*	ethanol	H	disc diffusion	n.d	n.d	-	[36]
*Salmonella enterica* ssp. *arizonae*	*F. laevis*	ethanol	AP	microdilution	n.d	77.66 ± 0.14	n.d	[41]
*Salmonella* *typhimurium*	*F. laevis*	EO	AP	disc diffusion	0.5	n.d	n.d	[43]
*Salmonella typhi*	*F. thymifolia*	methanol chloroform	Sh Sh	disc diffusion disc diffusion	n.d	n.d	6.0 10.5	[23]
*Pseudomonas* *aeruginosa*	*F. laevis*	EO	AP	disc diffusion	-	n.d	n.d	[43]
	*F. thymifolia*	methanol chloroform	Sh Sh	disc diffusion disc diffusion	n.d n.d	n.d n.d	6.0 8.1	[23]
	*F. thymifolia*	*n*-butanol ethyl acetate	AP	disc diffusion disc diffusion	n.d n.d	n.d n.d	12.0 7.0	[42]
	*F. hirsuta*	ethanol	H	disc diffusion	n.d	n.d	9.0	[36]
	*F. triandra*	ethanol water	AP	microdilution macrodilution	0.3	n.d	n.d	[74]
*Klebsiella* *oxytoca*	*F. thymifolia*	*n*-butanol ethyl acetate	AP	disc diffusion disc diffusion	n.d n.d	n.d n.d	9.0 10.0	[42]
*Enterobacter* *aerogenes*	*F. hirsuta*	ethanol	H	disc diffusion	n.d	n.d	-	[36]
*Morganella* *morganii*	*F. triandra*	ethanol-water	AP	microdilution macrodilution	0.15	n.d	n.d	[74]
**Fungi**								
*Candida albicans*	*F. thymifolia*	methanol chloroform	Sh Sh	disc diffusion disc diffusion	n.d n.d	n.d n.d	6.0 9.5	[23]
	*F. hirsuta*	ethanol	H	disc diffusion	n.d	n.d	12.0	[36]
*Rhizoctonia solani*	*F. pulverulenta*	EO	AP	well diffusion	n.d	n.d	12.25	[18]
*Penicillium* *simplicissimum*	*F. pulverulenta*	EO	AP	well diffusion	n.d	n.d	-	[18]
*Fusarium* *oxysporum*	*F. pulverulenta*	EO	AP	well diffusion	n.d	n.d	-	[18]
	*F. laevis*	EO	AP	disc diffusion	-	n.d	n.d	[43]
*Penicillium* *citrinum*	*F. pulverulenta*	EO	AP	well diffusion	n.d	n.d	-	[18]
*Fusarium* *fujikuroi*	*F. pulverulenta*	EO	AP	well diffusion	n.d	n.d	-	[18]
*Aspergillus niger*	*F. laevis*	EO	AP	disc diffusion	-	n.d	n.d	[43]
*Alternaria sp.*	*F. laevis*	EO	AP	disc diffusion	-	n.d	n.d	[43]
*Penicillium sp.*	*F. laevis*	EO	AP	disc diffusion	-	n.d	n.d	[43]
**Parasite**								
*Acanthamoeba castellanii* str. Neff.	*F. thymifolia*	*n*-butanol ethyl acetate	AP	modified Alamar Blue^®^	95.43 66.25	n.d n.d	n.d n.d	[42]
*Leishmania amazonensis*	*F. thymifolia*	*n*-butanol ethyl acetate	AP	modified Alamar Blue^®^	100.13 99.36	n.d n.d	n.d n.d	[42]
*Leishmania donovani*	*F. thymifolia*	*n*-butanol ethyl acetate	AP	modified Alamar Blue^®^	- -	n.d n.d	n.d n.d	[42]
*Trypanosoma cruzi*	*F. thymifolia*	*n*-butanol ethyl acetate	AP	modified Alamar Blue^®^	- -	n.d n.d	n.d n.d	[42]
*Schistosoma mansoni*	*F. hirsuta*	methanol	H	viability test	n.d	46.80/68.50	n.d	[31]
**Virus**								
HSV-1	*F. pulverulenta*	acetone methanol	AP AP	neutral red incorporation	n.d n.d	n.d n.d	489.5 486.2	[75]

Gram+: Gram-positive. Gram−: Gram-negative. HSV-1: *Herpes simplex* virus type 1. (-): no activity detected. n.d: not determined. AP: aerial parts. Sh: shoots. H: herb. EO: essential oil. MIC: minimum inhibitory concentration. MSI: microbial susceptibility index. IZ: inhibition zone. IC_50_: half maximal inhibitory concentration. LC_50_: 50% lethal concentration. LC_90_: 90% lethal concentration. EC_50_: half maximal effective concentration. Ref.: references.

#### 3.2.3. Neuroprotective Activity

Alzheimer’s disease (AD) is a neurodegenerative disease characterized by progressive and irreversible memory loss and other cognitive impairments. At the cellular level, AD is characterized by synaptic and neuronal loss, deposition of plaques made of β-amyloid peptide (Aβ) and the formation of fibrils in the brain made of tau-protein. Several data issued from genetic, neuropathological and biochemical studies have established the central role of the β-amyloid peptide (Aβ), which results from the cleavage of the so-called amyloid precursor protein (APP), a membrane glycoprotein [74,75]. However, its precise role in AD pathogenesis is still unclear.

One hypothesis suggests that oxidative damage in the brain may cause ROS generation in neurons, which in turn could potentiate the Aβ neurotoxicity and metabolism perturbation [76]. Therefore, limiting or inhibiting oxidative stress could be a way to treat AD. As various plants contain various antioxidant natural products, especially those of the *Frankenia* family (see Section 2.1.1 and Section 2.1.2), the neuroprotective properties of plants have been, and are, still being explored [77].

In order to determine whether *Frankenia* plant species may prevent Aβ-induced neuronal cell toxicity, neuroprotection tests were carried out. The neuroprotective potential of ethyl acetate fractions from *F. pulverulenta* shoots and roots was evaluated [19]. An Aβ(25–35)-induced cytotoxicity assay using pheochromocytoma-derived (PC12) cells was assessed. Both fractions remarkably prevented the cytotoxic response of Aβ(25–35) at levels around 57% and 80% at 100 and 200 μg/mL, respectively, compared with non-treated cells. At a higher concentration (300 μg/mL), the root fraction entirely counteracted the toxic effect of Aβ(25–35). Using the same process, the neuroprotective capacities of methanolic extracts from *F. thymifolia* and *F. pulverulenta* aerial parts were demonstrated in another study [39]. Both species exerted a powerful neuroprotective effect in a dose-dependent manner, and about 80% of the cell viability was restored at 100 μg/mL. Additionally, the ethyl acetate fractions from *F. thymifolia* shoots and roots demonstrated a strong neuroprotective effect on neuronal PC12 cells and totally counterbalanced the damaging effect of Aβ(25–35) at 25 and 50 μg/mL, respectively [21]. Most phenolics isolated from *F. pulverulenta* ethyl acetate fractions were shown to exhibit potent neuroprotective activities, particularly procyanidin dimers (**78**), which prevented Aβ-induced toxicity at levels close to 100% at 50 μM, while catechin (**74**) prevented it only at 70% at the same concentration, and quercetin (**62**) did not [19].

The strong capacity of *F. thymifolia* and *F. pulverulenta* extracts to inhibit Aβ(25–35) aggregation could be attributed to their significant antioxidant activities and phenolic contents. Various reports have indeed shown that phenolic substances may prevent neurodegenerative disorders, either by directly preventing the formation of Aβ fibril deposits in the brain [78] or by exhibiting protective effects through scavenging ROS [79]. Furthermore, a two-to-one complex between a polyphenol and the full Aβ peptide was observed by ESI-MS [78]. It was also reported that gallic acid (**1**), found in *F. thymifolia* roots, in its glucosylated form and the corresponding gallotannins effectively suppressed Aβ(25–35) aggregation in vitro [80]. Another study revealed that kaempferol-3-*O*-glucoside (**56**) presented a modest inhibitory effect on Aβ(25–35) aggregation, whereas kaempferol itself had a moderate effect. However, the reverse situation was observed with quercetin (**62**) and its 3′-*O*-glucoside (**72**), the latter exhibiting a good activity while the former had a modest one [81]. These results are quite surprising due to the structural similarity between these compounds (see Figure 4). Interestingly, the very similar hyperoside (**73**) significantly diminished Aβ-induced cytotoxicity and apoptosis by restoring Aβ-induced mitochondrial dysfunction [82]. Alternatively, it has been shown that caffeic acid (**30**), epigallocatechin (**75**) and its 3-*O*-gallate (**76**) exhibit a modest aggregation inhibition, and *p*-hydroxybenzoic acid (**11**) presents a moderate one, while the hydroxy derivatives of benzyl benzoate (**192**) exhibit interesting inhibition [83]. However, the latter have so far not been detected in *Frankenia* species.

Another approach to facing AD is to attempt to treat the synaptic and neuronal loss associated with AD. During the progression of AD, different types of neurons deteriorate, but the main loss occurs in forebrain cholinergic neurons, which play an important role in cognition. Therapies have thus been, and are still being, designed to reverse this cholinergic deficit. Cholinergic neurons rely on acetylcholine (ACh) as a neurotransmitter, which is hydrolyzed by acetylcholinesterase (AChE) in the synapse and to a lesser extent by the non-specific butyrylcholinesterase (BuChE) [84]. Furthermore, several studies have suggested that AChE can modulate APP processing in a way that enhances β-amyloid plaque deposition [85]. As a consequence, the inhibition of these enzymes is actively pursued. Various inhibitors have proven beneficial as a curative approach to AD, and a few are commercially available [84].

As some of the earliest inhibitors discovered were alkaloids issued from plants, plant extracts are now often evaluated as cholinesterase inhibitors. In *Frankenia* species, only a few have so far been evaluated. Interestingly, methanol extracts from *F. laevis* demonstrated significant AChE and BuChE inhibition (about 80% at 1 mg/mL) [13].

#### 3.2.4. Tyrosinase Inhibition Activity

Tyrosinase is a multipurpose copper-containing oxidase that participates in melanin production and enzymatic browning processes that happen in damaged fruits during post-harvest processing [86]. Natural substances are widely utilized in cosmetic formulations as tyrosinase inhibitors to cure skin hyperpigmentation, melasma and post-inflammatory hyperpigmentation [87]. They are also applied in the food industry to prohibit enzymatic browning action in injured vegetables [86].

The inhibition of tyrosinase by *F. laevis* shoot extracts (50% ethanol) was conducted by performing both the inhibition of l-tyrosine hydroxylation to l-3,4-dihydroxyphenylalanine (L-DOPA) (monophenolase) and that of L-DOPA oxidation to dopaquinone (diphenolase) [41]. A strong inhibition of monophenolase and diphenolase functions was achieved (IC_50_ = 730.43 and 123.62 μg/mL, respectively). In agreement with previous studies [88,89], the high levels of phenolic compounds, such as chlorogenic acid (**31**) and quercetin (**62**), in *F. laevis* extracts are probably responsible for the anti-tyrosinase effect, making this species a prospective source of natural skin-lightening agents and conservatives [86,87].

#### 3.2.5. Anti-Inflammatory Activity

Inflammation is induced by either external or internal causes. In the former, inflammation occurs in response to infection caused by microorganisms or to tissue injury. In the latter, cell death, cancer and other dysfunctions initiate a cascade of events leading to inflammation. In turn, various inflammatory mediators are produced, such as cytokines, chemokines, polyunsaturated fatty acids, etc., some acting as pro- and/or anti-inflammatory agents. The enzymes that are responsible for the generation of these inflammatory mediators, such as cyclooxygenase (COX), lipoxygenase (LOX) and hyaluronidase, are the major targets of anti-inflammatory therapies and a number of drugs have been developed [90]

For such common anti-inflammatory activity, the use of plants has been known since antiquity and is still applied. Although traditional medicines provide numerous anti-inflammatory extracts or plant parts, this activity is still being explored and remains one of the most sought-after bioactivities from plants [91].

The anti-inflammatory capacity of ethanolic and soxhlet extracts obtained from *F. triandra* aerial parts was evaluated [34]. The inhibition of LOX and COX2 capacities was assessed on the basis of the enzymatic oxidation of linoleic acid to the corresponding hydroperoxide and prostaglandin measurement, respectively. The extracts displayed a satisfactory ability to prevent LOX (IC_50_ = 134.5 ± 12.9 and 117.8 ± 1.8 μg/mL, respectively) and COX2 (54% and 50% inhibition, respectively) actions. Hence, it is thought that these inhibition values are high for a crude extract [92]. The authors have also examined the hyaluronidase activity by measuring the quantity of generated *N*-acetyl glucosamine (NAGA) [34]. Both soxhlet and ethanolic extracts demonstrated a high degree of inhibition, but the soxhlet extract was three times more effective than the ethanolic extract (IC_50_ = 146.3 ± 4.3 and 412.2 ± 8.9 μg/mL, respectively) as compared to the commercial anti-inflammatory, indomethacin (IC_50_ = 502.0 ± 7.1 μg/mL), and the control sample, quercetin (IC_50_ = 340.0 ± 12.0 μg/mL).

Numerous studies have shown a strong correlation between inflammation and oxidative species production. Consequently, plants with antioxidant capabilities frequently have anti-inflammatory characteristics [93].

#### 3.2.6. Carbonic Anhydrase II Inhibition Activity

Carbonic anhydrase II (CA-II) belongs to the carbonic anhydrase family of enzymes, which are zinc metalloenzymes that catalyze the reversible conversion of carbon dioxide (CO_2_) to bicarbonate (HCO_3_) and a proton (H^+^) [94]. In addition to their key roles in transporting CO_2_ and maintaining acid–base balance, the 16 human carbonic anhydrases are also involved in several essential physiological processes, and, thus, their dysregulated expression and/or abnormal activity have important pathological consequences. For example, CA-II is mainly involved in the regulation of bicarbonate concentration in the eyes, and is thus linked to glaucoma, but also expressed in malignant brain tumors and renal, gastritis and pancreatic carcinomas. CA-II and other CAs are therefore interesting therapeutic targets for the treatment of related diseases. CA-II inhibitors are, for example, used in the treatment of several illnesses, including glaucoma, idiopathic intracranial hypertension, altitude sickness, congestive heart failure and epilepsy [95,96,97,98].

In order to look for some activity in such health problems, the EO extracted from the aerial parts of *F. pulverulenta* was screened against the CA-II enzyme. The experiment was done at a micromolar level using acetazolamide as a standard inhibitor (IC_50_ = 18.2 ± 1.2 μM). The EO demonstrated a substantial and spectacular CA-II inhibition effect (IC_50_ = 101.5 ± 2.35%) and might have application in the management of CA-related disorders [18].

#### 3.2.7. Antidiabetic Activity

In type 2 diabetic patients postprandial hyperglycemia occurs because the peak insulin release is delayed, and levels are thus insufficient to control the accelerated blood glucose elevation. Such hyperglycemic spikes induce inflammatory reactions, oxidative stress and endothelial dysfunction, which in turn increase the occurrence of cardiovascular diseases.

To reduce postprandial hyperglycemia, the most common type 2 diabetes preventive therapy involves decreasing carbohydrate digestibility by blocking two important hydrolyzing enzymes, specifically, α-amylase and α-glucosidase [99].

The methanol and dichloromethane extracts of *F. laevis* were investigated for their capacity to inhibit α-glucosidase and α-amylase enzymes [13] using a standard in vitro inhibition assay [100]. The extracts showed a marked α-glucosidase inhibition (EC_50_ = 1.02 ± 0.01 mg/mL and 0.52 ± 0.04 mg/mL, respectively) compared to the positive control, acarbose (EC_50_ = 3.14 ± 0.23 mg/mL). On the other hand, the extracts had no significant effect on α-amylase activity.

Abundant in *F. laevis* extracts, linoleic acid (**174**) and its derivatives, as well as loliolide (**107**), isololiolide (**106**) and dihydroactinidiolide (**108**), were found to have a strong inhibitory effect on α-glucosidase. Their higher abundance in the dichloromethane extract may explain the anti-α-glucosidase activity of the *F. laevis* extracts. In addition, the antioxidant properties of these extracts may also help to decrease the incidence of diabetes complications related to oxidative stress, specifically microvascular and cardiovascular issues [101].

#### 3.2.8. Anticancer Activity

Prior to human usage, substances or chemicals must undergo rigorous safety evaluations. Cytotoxic tests using various human cell lines are often performed to assess the potential toxicity of different substances in vitro [102]. The 3-(4,5-dimethylthiazol-2-yl)-2, 5-diphenyltetrazolium bromide (MTT) test, a colorimetric approach that measures cell metabolic activity, is one of the most frequently used methods to determine how a substance affects cellular viability [102]. On the other hand, cytotoxicity could be useful to control tumor cell proliferation and thus treat cancers.

For the latter purpose, the anticancer and antiproliferative activities of extracts from the aerial parts of *F. laevis* were investigated against human hepatocarcinoma cells (HepG2) [13]. Sea heath dichloromethane extract showed potential anti-HepG2 activity (EC_50_ = 52.1 μg/mL). In contrast, methanol extract did not present significant cytotoxicity. This difference could be ascribed to the high content of certain metabolites and fatty acids in the dichloromethane extract.

It has indeed been reported that fatty acids, especially linoleic acid (**174**) which is abundant in this extract, have shown chemoprotective effects [103,104]. Furthermore, the monoterpenes loliolide (**107**) and isololiolide (**106**), also abundant in this extract, are known for their strong cytotoxic activities on HepG2 cells [105,106], as is dihydroactinidiolide (**108**) on human lung carcinoma cells (A549) [107] and human breast cancer cells [106,108]. The phytohormone oxophytodienoic acid (**204**) also present in this extract is also known for its cytotoxic activity on human breast cancer cells [109].

A similar study on a large series of halophyte plants, including both *F. laevis* and *F. pulverulenta,* has been performed [17]. The viability of four cancer cell types, including the same HepG2 cell line, was evaluated, and the *F. laevis* extract was found to significantly decrease it (71%), while *F. pulverulenta* did not.

Due to the abundance of the active compounds mentioned above, the *F. laevis* dichloromethane extract may represent an interesting natural alternative for treating some cancers. Furthermore, the natural products probably responsible for these antitumor activities could become promising candidates for new antitumor drugs.

#### 3.2.9. Insecticidal Activity

The control of insect proliferation and of the so-induced destruction of agricultural plants is usually achieved with synthetic insecticides. However, their intensive and uncontrolled utilization has led to the development of resistance in insects and to various environmental damages. Although a few insecticides are issued from plants, such as pyrethrenoids, plants may provide potentially safer alternatives to the currently used insect-control agents.

In this context, petroleum ether and chloroformic and ethyl acetate extracts obtained from the aerial parts of *F. laevis* were evaluated for their antifeedant, toxic and insect growth inhibition activities against larvae and adults of the confused flour beetle *Tribolium confusum* [110]. At a concentration of 1%, the petroleum ether extract demonstrated moderate antifeedant properties. At the same concentration, the tested extracts considerably induced larval mortality (up to 97% inhibition with the ethyl acetate extract), while adult toxicity did not surpass 33%. Furthermore, the *F. laevis* extracts inhibited feeding, exhibited high toxicity and greatly affected the development of *Tribolium confusum* larvae when used at a dose of 1%. Therefore, this halophyte plant seems to have great potential for pest control; it would be worth identifying the compound(s) responsible for the interesting insecticidal activity of these extracts even at low concentrations [110].

## 4. Conclusions

In this review, we have described a series of *Frankenia* plant species known for their role in traditional medicine. These plants are indicated for the treatment of a variety of illnesses, including diarrhea, respiratory issues and wounds. The corresponding phytochemical investigations have been collected here and analyzed. These data revealed that these *Frankenia* species produce a wide range of interesting metabolites. They contain relatively high levels of specific substances, such as phenolics, flavonoids and terpenoids, as well as various fatty acids, as such or as derivatives, and alkanes. Some alkaloids and steroids have also been identified, but only a few lignans and coumarins have so far been observed.

Furthermore, the corresponding biological investigations have also been collected when available and the results have been interpreted as much as possible in terms of the chemical content of each extract or part of the plants. Interestingly, these in vitro studies revealed a variety of biological activities, from the classical antioxidant effects and the related anti-inflammatory activity to enzyme inhibitions, neuroprotection, anti-diabetic and anti-tumor activities, as well as insecticidal properties. All these bioactivities are obviously linked to the application of *Frankenia* plants in traditional medicine. However, the molecular mechanisms of these biological effects correlated to the chemicals recovered from *Frankenia* species remain unclear.

As shown here, the value of plants as sources of bioactive natural substances resides not only in their insecticidal, pharmacological or chemotherapeutic effects but also in their roles in the development of novel drugs [111]. However, the number of higher plant species on earth is estimated to be between 250,000 and 500,000, from which only 15% have been evaluated phytochemically and only 6–7% have been screened for biologic activity [112]. It is thus worth looking at more plants for their chemical profile and their biological activity. Unfortunately, only six species of the *Frankenia* genus were investigated in detail for their chemical composition and/or pharmacological activities.

In summary, research on *Frankenia* species is still in its infancy and needs to be developed further, in order to discover novel bioactive compounds and better understand the correlation between the identified natural substances and the corresponding biological activity. Additionally, future research should also expand on in vivo studies and clinical trials to learn more about the potential modes of action in human metabolic disorders and illnesses.

## Figures and Tables

**Table 1:** Typical *Frankenia* species, their common and synonym names, from which phytochemical profiles have been established, and their geographic distribution.

Species Name	Synonym	Common Name	Distribution
*Frankenia laevis* L. ^a^ 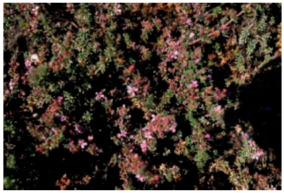	*Hypericopsis* Boissier. [12] *Frankenia canescens* Presl.	Sea heath [13]	Portugal, Spain, France [13] Algeria, Morocco, Tunisia
	*Frankenia intermedia* Costa.		Egypt [14], Iran [15]
	*Frankenia laevis* Habl. ex Bieb.		
		
*Frankenia pulverulenta* L. ^b^ 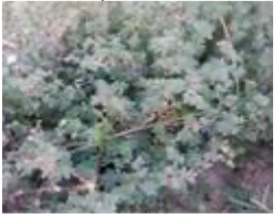	*Frankenia nodiflora* Lam. [16]	European sea heath [17] Annual sea heath [16]	Tunisia [18,19]
		
*Frankenia thymifolia* Desf. ^c^ 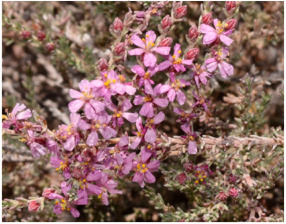	*Frankenia reuteri* Boiss. [20]	Thyme sea heath	North Africa [21,22,23] Oman [18], Iraq [24], Iran [25]
			Spain [26], Portugal [17], England [27]
*Frankenia hirsuta* L. ^d^ 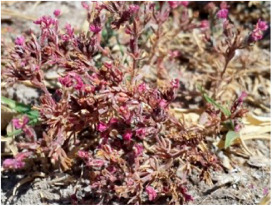	*Frankenia aucheri* Jaub. and Spach [12] *Frankenia hirsuta* L. var. *erecta* Boiss.	Millaih, Shuwaiwa [15] Hairy sea heath [28]	Saudi Arabia [18]
	*Frankenia salsuginea*		Turkey [29]
	*Frankenia hispida* D. C.		Greece [30], Egypt [31], Iraq [32], Iran [33]
*Frankenia triandra* J. Rémy ^c^ 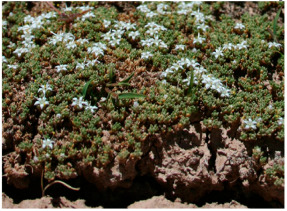		Yareta Yaretilla [34]	Argentina, Chile Bolivia [34]
*Frankenia foliosa*		Leafy sea heath	Australia [35]

^a^ Picture adapted from [13]; ^b^ Picture adapted from [24]; ^c^ Picture adapted from the website of the Royal Botanical Kew Garden, London, https://powo.science.kew.org/ accessed on 6 February 2024; ^d^ Picture adapted from [36].

**Table 2:** Phenolics from *Frankenia* species.

Compound	Substituents	Species	References
(**1**) gallic acid	R1 = H; R2 = H; R3 = H; R4 = H	*F. laevis,* *F. pulverulenta, F. thymifolia*	[19,21,39,40,41]
(**2**) gallic acid-3-methyl ether	R1 = R2 = H; R3 = H; R4 = CH_3_	*F. laevis*	[14,40]
(**3**) gallic acid-3-methyl ether-5-sodium sulphate	R1 = R3 = H; R2 = SO_3_Na; R4 = CH_3_	“ ^a^	[14]
(**4**) gallic acid sulfate	R1 = R2 = R3 = H; R4 = SO_3_H	“	[13]
(**5**) methyl gallate-3,4-dimethyl ether	R1 = R3 = R4 = CH_3_; R2 = H	*F. thymifolia*	[42]
(**6**) 3-*O*-methylgallic acid-5-*O*-sulfate	R1 = R3 = H; R2 = CH_3_; R4 = SO_3_H	*F. laevis*	[13]
(**7**) 4-*O*-methylgallic acid	R1 = R2 = H; R3 = CH_3_; R4 = SO_3_H	“	“ ^b^
(**8**) trimethylgallate (eudesmic acid)	R1 = H; R2 = R3 = R4 = CH_3_	*F. hirsuta*	[36]
(**9**) 4,5-dimethoxy-3-hydroxybenzoic acid methyl ester	R1 = R3 = R4 = CH_3_; R2 = H;	*F. thymifolia*	[22]
(**10**) salicylic acid	R1 = OH; R2 = R3 = R4 = H	“	[23]
(**11**) *p*-hydroxybenzoic acid	R1 = R2 = R4 = H; R3 = OH	“	[21]
(**12**) 2,5-dihydroxybenzoic acid	R1 = R4 = OH; R2 = R3 = H;	“	[23]
(**13**) vanillic acid	R1 = R2 = H; R3 = OH; R4 = OCH_3_	“	[23]
(**14**) ellagic acid	R1 = R2 = R3 = R4 = H	*F. laevis*	[13,40]
(**15**) 3-*O*-methylellagic acid	R1 = CH_3_; R2 = R3 = R4 = H	“	“
(**16**) 3-*O*-methylellagic acid-4-*O*-sulfate	R1 = CH_3_; R2 = SO_3_H; R3 = R4 = H	“	[13]
(**17**) 3,3′-di-*O*-methylellagic acid-4-*O*-sulfate	R1 = R4 = CH_3_; R2 = SO_3_H; R3 = H;	“	“
(**18**) 3,3′-di-*O*-methylellagic acid	R1 = R4 = CH_3_; R2 = R3 = H;	“	[13,14]
(**19**) ellagic acid-3-methyl ether	R1 = CH_3_; R2 = R3 = R4 = H	“	[14,40]
(**20**) ellagic acid-3-methyl ether-4′-sodium sulphate	R1 = R3 = H; R2 = SO_3_Na; R4 = CH_3_	“	[40]
(**21**) ellagic acid-3,3′-dimethyl ether-4-sodium sulphate	R1 = R4 = CH_3_; R2 = H; R3 = SO_3_Na;	“	[14]
(**22**) ellagic acid-3,3′-dimethyl ether-4,4′-di-sodium sulphate	R1 = R4 = CH_3_; R2 = R3 = SO_3_Na	“	“
(**23**) ellagic acid-3-methyl ether-4-sodium sulphate	R1 = R2 = H; R3 = SO_3_Na; R4 = CH_3_	“	“
(**24**) 3,3′,4-tri-*O*-methylellagic acid	R1 = R2 = R4 = CH_3_; R3 = H	“	[13]
(**25**) 3,3′,4-tri-*O*-methylellagic acid-4′-*O*-sulfate	R1 = R2 = R4 = CH_3_; R3 = SO_3_H;	“	“
(**26**) 3-*O*-methylellagic acid-4′-*O*-glucoside	R1 = R3 = H; R2 = glucose; R4 = CH_3_	“	“
(**27**) 3,3′-di-*O*-methylellagic acid-4-*O*-glucoside	R1 = CH_3_; R2 = glucose; R3 = H;R4 = CH_3_	“	“
(**28**) *E*-cinnamic acid	R1 = H; R2 = H; R3 = H; R4 = H; R5 = OH	*F. thymifolia*	[23]
(**29**) *E*-2-hydroxycinnamic acid	R1 = R2 = R3 = H; R4 = R5 = OH	“	“
(**30**) caffeic acid	R1 = R2 = R5 = OH; R3 = R4 = H	“	[39]
(**31**) chlorogenic acid	R1 = R2 = OH; R3 = R4 = H; R5 = 1,3,4-trihy droxycyclohexane-1-carboxylic acid	“	[23,39]
(**32**) sinapic acid	R1 = OCH_3_; R2 = OH; R3 = OCH_3_; R4 = H; R5 = OH	“	[39]
(**33**) caffeic acid sulfate	R1 = OSO_3_H; R2 = R5 = OH; R3 = R4 = H;	*F. laevis*	[13]
(**34**) *p*-coumaric acid	R1 = R3 = R4 = H; R2 = R5 = OH	“	[41]
(**35**) *p*-coumaric acid 4-*O*-sulfate	R1 = R3 = R4 = H; R2 = OSO_3_H; R5 = OH	“	[13]
(**36**) ferulic acid 4-*O*-sulfate	R1 = R3 = OCH3; R2 = OSO_3_H; R4 = H; R5 = OH	“	“
(**37**) coumaroyl hexose sulfate	R1 = R3 = R4 = H; R2 = OH; R5 = OCH_2_CHOSO_3_H(CHOH)_3_CH_2_OH	“	“
(**38**) caffeoyl pentose sulfate	R1 = OH; R2 = OH; R3 = H; R4 = H; R5 = OCH_2_CHOSO_3_H(CHOH)_2_CH_2_OH	“	“
(**39**) caffeoyl hexose sulfate	R1 = OH; R2 = OH; R3 = H; R4 = H; R5 = OCH_2_CHOSO_3_H(CHOH)_3_CH_2_OH	“	“
(**40**) feruloyl hexose sulfate	R1 = OCH_3_; R2 = OH; R3 = H; R4 = H; R5 = OCH_2_CHOSO_3_H(CHOH)_3_CH_2_OH	“	“
(**41**) *N*-*cis*-feruloyltyramine	R1 = R3 = R4 = H; R2 = OH; R5 = 4-(2-aminoethyl)phenol	“	“
(**42**) acetophenone-4-methylether	R = H	“	[14]
(**43**) acetophenone-4-methylether-2-sodium sulfate	R = SO_3_Na	“	[14]
(**44**) catechol	R1 = R2 = R5 = H; R3 = R4 = OH;	*F. pulverulenta*	[39]
(**45**) resorcinol	R1 = R3 = OH; R2 = R4 = R5 = H		“
(**46**) hydroxytyrosol	R1 = CH_2_CH_2_OH; R2 = R5 = H; R3 = R4 = OH;	*F. thymifolia*	[21]
(**47**) butenylpyrocatechol sulfate	R1 = OSO_3_H; R2 = OH; R3 = Butenyl; R4 = R5 = H	*F. laevis*	[13]
(**48**) butanoylpyrocatechol sulfate	R_1_ = OSO_3_H; R_2_ = OH; R_3_ = CO(CH_2_)_2_CH_3_; R_4_ = R_5_ = H	“	“
(**49**) eugenol	R1 = CH_2_CHCH_2_; R2 = R5 = H; R3 = OCH_3_; R4 = OH	“	[43]
(**50**) 1,3-dithian-2-yl(phenyl)methanone	R1 = (1,3-dithian-2-yl)oxomethyl; R2 = R3 = R4 = R5 = H	*F. hirsuta*	[32]
(**51**) 3-tertbutyl-5-chloro-2-hydroxybenzo phenone	R1 = CO(phenyl); R2 = OH; R3 = C(CH_3_)_3_; R4 = H; R5 = Cl	*F. pulverulenta*	[24]
(**52**) posthumulone		*F. thymifolia*	[21]
(**53**) α-tocopherol (vitamin E)		*F. hirsuta*	[36]

^a^ The mark “ indicates that the same species as above is concerned. ^b^ The mark “ indicates that the same reference as above is concerned.

**Table 3:** Flavonoids from *Frankenia* species.

Compound	Substituents	Species	References
(**54**) kaempferol sulfate	R1 = SO_3_H; R2 = H	*F. laevis*	[13]
(**55**) kaempferol-3-sodium sulfate	R1 = SO_3_Na; R2 = H	“ ^a^	[40]
(**56**) kaempferol-3-*O*-glucoside	R1 = glucosyl; R2 = H	*F. thymifolia*	[21]
(**57**) kaempferol-3-*O*-rutinoside	R1 = rutinosyl; R2 = H	*F. pulverulenta*	[39]
(**58**) kaempferol-7-sodium sulfate	R1 = H; R2 = SO_3_Na	*F. laevis*	[40]
(**59**) kaempferol-7-bisulfate	R1 = H; R2 = SO_3_H	*F. pulverulenta*	[27]
(**60**) kaempferol-3,7-disodium sulfate	R1 = SO_3_Na; R2 = SO_3_ Na	*F. laevis*	[40]
(**61**) kaempferol-7-bisulfate-3-glucuronide	R1 = glucuronide; R2 = SO_3_H	*F. pulverulenta*	[27]
(**62**) quercetin	R1 = H; R2 = H; R3 = H	*F. thymifolia*	[42]
(**63**) quercetin-3-*O*-methyl ether	R1 = H; R2 = CH_3_; R3 = H	“	“ ^b^
(**64**) quercetin-3-sodium sulfate	R1 = H; R2 = SO_3_Na; R3 = H	*F. laevis*	[40]
(**65**) quercetin-3-bisulfate	R1 = H; R2 = SO_3_H; R3 = H	*F. pulverulenta*	[27]
(**66**) quercetin-7-bisulfate	R1 = H; R2 = H; R3 = SO_3_H	“	“
(**67**) quercetin-7-sodium sulfate	R1 = R2 = H; R3 = (SO_3_)Na	*F. laevis*	[40]
(**68**) quercetin 7-bisulfate-3-glucuronide	R1 = H; R2 = glucuronide; R3 = SO_3_H	*F. pulverulenta*	[27]
(**69**) quercetin-3,7-disodium sulfate	R1 = H; R2 = SO_3_ Na; R3 = SO_3_Na	*F. laevis*	[40]
(**70**) quercetin-3′-bisulfate	R1 = (SO_3_H) Na; R2 = R3 = H	*F. pulverulenta*	[27]
(**71**) quercetin-3′-*O*-β-galactopyranoside	R1 = galactopyranosyl; R2 = R3 = H	*F. thymifolia*	[42]
(**72**) quercetin-3′-*O*-β-glucopyranoside	R1 = glucopyranosyl; R2 = R3 = H	“	“
(**73**) quercetin-3-*O*-galactoside (hyperoside)	R1 = R3 = H; R2 = galactosyl;	“	[21]
(**74**) catechin	R1 = R2 = H	*F. laevis,* *F. pulverulenta*, *F. thymifolia*	[19,23,39,41]
(**75**) epigallocatechin	R1 = OH; R2 = H	*F. pulverulenta*	[39]
(**76**) epigallocatechino-3-gallate	R1 = OH; R2 = gallate	*F. thymifolia*	[23]
(**77**) prodelphinidin B-4		“	[21]
(**78**) procyanidin (dimer 1,2 and 3)		*F. pulverulenta*	[19]
(**79**) isorhamnetin-7-bisulfate	R1 = H; R2 = SO_3_H	“	[27]
(**80**) isorhamnetin-7-bisulfate-3-glucuronide	R1 = glucuronide; R2 = SO_3_H	“	“
(**81**) isorhamnetin-*O*-pentosylhexoside	R1 = pentosyl-hexoside; R2 = H	*F. laevis*	[13]
(**82**) naringenin		*F. thymifolia*	[42]
(**83**) luteolin-7-*O*-glucoside		*F. pulverulenta, F. thymifolia*	[21,39]

^a^ The mark “ indicates that the same species as above is concerned. ^b^ The mark “ indicates that the same reference as above is concerned.

**Table 4:** Lignans and coumarins from *Frankenia* species.

Compound	Substituents	Species	References
(**84**) lyoniresinol sulfate		*F. laevis*	[13]
(**85**) lariciresinol		“ ^a^	“ ^b^
(**86**) pinoresinol	R = H	*F. thymifolia*	[21]
(**87**) pinoresinol-4-sulfate	R = OSO_3_H	“	[22]
(**88**) coumarin		*F. pulverulenta*	[39]

^a^ The mark “ indicates that the same species as above is concerned. ^b^ The mark “ indicates that the same reference as above is concerned.

**Table 5:** Alkaloids from *Frankenia* species.

Compound	Substituents	Species	References
(**89**) *S*-methyl, *N*-(2-methyl-3-oxobutyl) dithiocarbamate		*F. pulverulenta*	[24]
(**90**) 1,8-di-(4-nitrophenylmethyl)-3,6-diazahomoadamantan-9-one		“ ^a^	“ ^b^
(**91**) pyrrolizin-1,7-dione-6-carboxylic acid, methyl ester		“	“
(**92**) *N*-methyl, *N*-4-[1-(pyrrolidinyl)-2-butynyl] formamide		“	“
(**93**) *N*-cyclooct-4-enyl acetamide		“	“
(**94**) 2-(2-methyl-propenyl)-cyclohexanone oxime		“	“
(**95**) 1-propyl-3,6-diaza homoadamantan-9-ol	R1 = H; R2 = (CH_2_)_2_CH_3_	“	“
(**96**) 1,8-diethyl-3,6-diaza homoadamantan-9-ol	R1 = R2 = CH_2_CH_3_	“	“
(**97**) 16,17-didehydrocuran	R1 = R2 = R3 = H	“	“
(**98**) (19*S*)-16,17-didehydrocuran-19,20-diol	R1 = H; R2 = R3 = OH	“	“
(**99**) 1-acetyl-20α-hydroxy-16-methylene strychnane	R1 = COCH_3_; R2 = OH; R3 = H	“	“
(**100**) dasycarpidan-1-methanol acetate		“	“
(**101**) 2,7-diphenyl-1,6-dioxopyridazino [4,5:2′,3′]pyrrolo [4′,5′-d]pyridazine		“	“
(**102**) dihydrotecomanine		*F. pulverulenta*, *F. hirsuta*	[24,32]
(**103**) 2-acetylamino-3-hydroxypropionic acid		*F. aucheri (irsute)*	[32]
(**104**) pterin-6-carboxylic acid		“	“
(**105**) 18,19-didehydro-10-methoxycorynan-17-ol, acetate		“	“

^a^ The mark “ indicates that the same species as above is concerned. ^b^ The mark “ indicates that the same reference as above is concerned.

**Table 6:** Terpenoids from *Frankenia* species.

Compound	Substituents	Species	References
(**106**) isololiolide	R = OH	*F. laevis*	[13]
(**107**) loliolide	R = OH	“ ^a^	“ ^b^
(**108**) dihydroactinidiolide	R = H	“	“
(**109**) α-pinene		*F. pulverulenta*	[18]
(**110**) phytol	R = OH	*F. laevis*	[43]
(**111**) (*E*)-phytyl acetate	R = OCOCH_3_	“	“
(**112**) isophytol		“	“
(**113**) (*E*,*E*,*E*)-geranylgeraniol		*F. pulverulenta*	[18]
(**114**) gibberellic acid		“	[24]
(**115**) caryophyllene oxide		*F. laevis*	[43]
(**116**) (*E*)-nerolidol		“	“
(**117**) (*E*,*E*)-farnesol	R = CH_2_OH	“	“
(**118**) (*E*,*E*)-farnesal	R = CHO	“	“
(**119**) (*E*,*E*)-farnesyl acetate	R = CH_2_OCOCH_3_	“	“
(**120**) α-copaene-11-ol		*F. pulverulenta*	[18]
(**121**) ledol		“	“
(**122**) α-cadinol		“	“
(**123**) *tau*-cadinol		“	“
(**124**) torreyol		“	“
(**125**) 6-epi-shyobunol		*F. hirsuta*	[32]
(**126**) germacrene D		*F. laevis*	[43]
(**127**) calarene		“	“
(**128**) α-copaene		*F. pulverulenta*	[18]
(**129**) β-humulene		“	“
(**130**) α-selinene		“	“
(**131**) β-selinene		“	“
(**132**) ledene		“	“
(**133**) δ-cadinene		“	“
(**134**) γ-cadinene		*F. laevis*, *F. pulverulenta*	[18,43]
(**135**) (*E*)-β-caryophyllene		“	“
(**136**) allo-aromadendrene		“	“

^a^ The mark “ indicates that the same species as above is concerned. ^b^ The mark “ indicates that the same reference as above is concerned.

**Table 7:** Steroids from *Frankenia* species.

Compound	Substituents	Species	References
(**137**) β-5,6-secosteroid	R = Et	*F. foliosa*	[35]
(**138**) 5-oxo-5,6-seco-3-cholesten-6-oic acid	R = H	“ ^a^	“ ^b^
(**139**) vitamin D (9,10-secosteroids)		“	“
(**140**) brassinolide (6,7-secosteroids)		“	“
(**141**) eringiacetal A		“	“
(**142**) ethyl iso-allocholate		*F. pulverulenta*	[24]
(**143**) 2-(3-acetoxy-4,4,14 trimethylandrost-8-en-17-yl)propanoic acid		“	“
(**144**) γ-sitosterol		*F. hirsuta*	[36]

^a^ The mark “ indicates that the same species as above is concerned. ^b^ The mark “ indicates that the same reference as above is concerned.

**Table 8:** Alkanes and alkenes from *Frankenia* species, corresponding to the global formula below.

Compound	Substituents 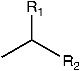	Species	References
(**145**) heptadecane	R1 = H; R2 = (CH_2_)_14_CH_3_	*F. laevis*	[43]
(**146**) tricosane	R1 = H; R2 = (CH_2_)_20_CH_3_	“ ^a^	“ ^b^
(**147**) tetracosane	R1 = H; R2 = (CH_2_)_21_CH_3_	*F. laevis, F. pulverulenta*	[18,43]
(**148**) pentacosane	R1 = H; R2 = (CH_2_)_22_CH_3_	“	“
(**149**) hexacosane	R1 = H; R2 = (CH_2_)_23_CH_3_	*F. laevis*	[43]
(**150**) heptacosane	R1 = H; R2 = (CH_2_)_24_CH_3_	“	“
(**151**) octacosane	R1 = H; R2 = (CH_2_)_25_CH_3_	“	“
(**152**) nonacosane	R1 = H; R2 = (CH_2_)_26_CH_3_	“	“
(**153**) triacontane	R1 = H; R2 = (CH_2_)_27_CH_3_	“	“
(**154**) docosane	R1 = H; R2 = (CH_2_)_19_CH_3_	*F. pulverulenta*	[18]
(**155**) *n*-heneicosane	R1 = H; R2 = (CH_2_)_18_CH_3_	*F. laevis*	“
(**156**) 2-methyloctacosane	R1 = CH_3_; R2 = (CH_2_)_25_CH_3_	“	“
(**157**) hentriacontane	R1 = H; R2 = (CH_2_)_28_CH_3_	“	“
(**158**) pentatriacontane	R1 = H; R2 = (CH_2_)_32_CH_3_	“	“
(**159**) eicosane	R1 = H; R2 = (CH_2_)_17_CH_3_	*F. hirsuta*	[36]
(**160**) 1-docosene	R1 = H; R2 = (CH_2_)_18_CHCH_2_	*F. laevis*	[43]
(**161**) 1-nonade-cene	R1 = H; R2 = (CH_2_)_15_CHCH_2_	*F. hirsuta*	[36]

^a^ The mark “ indicates that the same species as above is concerned. ^b^ The mark “ indicates that the same reference as above is concerned.

**Table 9:** Fatty acids and esters from *Frankenia* species, corresponding to the global formula below.

Compound	Substituents 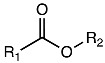	Species	References
(**162**) caproic acid	R1 = (CH_2_)_4_CH_3_; R2 = H	*F. laevis*	[43]
(**163**) caprylic acid	R1 = (CH_2_)_6_CH_3_; R2 = H	*F. hirsuta*	[32]
(**164**) pelargonic acid	R1 = (CH_2_)_7_CH_3_; R2 = H	“ ^a^	“ ^b^
(**165**) lauric acid	R1 = (CH_2_)_10_CH_3_; R2 = H	*F. laevis*, *F. thymifolia*	[23,43]
(**166**) myristic acid	R1 = (CH_2_)_12_CH_3_; R2 = H	*F. thymifolia*	[23]
(**167**) palmitic acid	R1 = (CH_2_)_14_CH_3_; R2 = H	*F. hirsuta*, *F. laevis*, *F. pulverulenta*, *F. thymifolia*	[23,24,36,43]
(**168**) thapsic acid	R1 = (CH_2_)_14_COOH; R2 = H	*F. laevis*	[43]
(**169**) stearic acid	R1 = (CH_2_)_16_CH_3_; R2 = H	*F. hirsuta*, *F. thymifolia*	[23,32,36]
(**170**) behenic acid	R1 = (CH_2_)_20_CH_3_; R2 = H	“	[23,36]
(**171**) lignoceric acid	R1 = (CH_2_)_22_CH_3_; R2 = H	*F. hirsuta*	[36]
(**172**) oleic acid	R1 = (*Z*)-heptadec-8-enyl; R2 = H	*F. hirsuta*, *F. thymifolia*	[23,36]
(**173**) elaïdic acid	R1 = (*E*)-heptadec-8-enyl; R2 = H	*F. thymifolia*	[23]
(**174**) linoleic acid	R1 = (8*Z*,11*Z*)-heptadeca-8,11-dienyl; R2 = H	*F. hirsuta*, *F. thymifolia*	[23,36]
(**175**) α-linolenic acid	R1 = (8*Z*,11*Z*,14*Z*)-heptadeca-8,11,14-trienyl; R2 = H	*F. thymifolia*	[23]
(**176**) gamolenic acid	R1 = (5*Z*,8*Z*,11*Z*)-hexadeca-5,8,11-trienyl; R2 = H	*F. pulverulenta*	[24]
(**177**) hydroxyoctadecadienoic acid	R1 = (1*E*,3*E*)-1-hydroxyheptadec-3-enylidene; R2 = H	*F. laevis*	[43]
(**178**) malyngic acid	R1 = (8*S*, 9*E*,11*R*,12*R*,13Z)-8,11,12-trihydroxyheptadeca-9,13-dienyl; R2 = H	“	[13]
(**179**) methyl *cis*-12,13-epoxyoctadecanoate	R1 = *cis*-11,13-epoxyheptadecyl; R2 = CH_3_	*F. hirsuta*	[32]
(**180**) methyl palmitate	R1 = pentadecyl; R2 = CH_3_	*F. laevis*	[43]
(**181**) methyl linoleate	R1 = (8*Z*,11*Z*)-heptadeca-8,11-dienyl; R2 = CH_3_	“	“
(**182**) methyl 12,15-octadecadiynoate	R1 = heptadeca-11,14-diynyl; R2 = CH_3_	*F. hirsuta*	[32]
(**183**) methyl-11,13-dihydroxytetradec-5-ynoate	R1 = 10,11-dihydroxytridec-4-ynyl; R2 = CH_3_	*F. pulverulenta*	[24]

^a^ The mark “ indicates that the same species as above is concerned. ^b^ The mark “ indicates that the same reference as above is concerned.

**Table 10:** Miscellaneous compounds isolated from *Frankenia* species.

Compound	Substituents	Species	References
(**184**) 5,7-dodecadiyn-1,12-diol		*F. pulverulenta*	[24]
(**185**) hexadecan-1-ol		*F. laevis*	[43]
(**186**) 3-*O*-methyl-d-glucose		*F. pulverulenta, F. hirsuta*	[24,32]
(**187**) 6-acetyl-α-d-mannose		*F. hirsuta*	[32]
(**188**) 6-acetyl-β-d-mannose		*F. pulverulenta*	[24]
(**189**) α-d-glucopyranosyl-(1->3)-β-d-fructofuranosyl β-d-glucopyranoside		“ ^a^	“ ^b^
(**190**) 4-*O*-(β-d-galactopyranosyl)-β-d-glucopyranose		“	“
(**191**) desulphosinigrin		*F. hirsuta*	[32]
(**192**) benzyl benzoate	R = phenyl	“	“
(**193**) benzyl cinnamate	R = styryl	“	“
(**194**) mesitylene		“	[36]
(**195**) 1,2,3,4,5,7-hexamethoxynaphthalene		*F. thymifolia*	[22]
(**196**) pheophytin A		*F. laevis*	[13]
(**197**) [(hexadecyloxy)methyl]oxirane		*F. hirsuta*	[32]
(**198**) 8a-methyl-4*H*,5*H*-tetrahydropyrano[4,3-d]-1,3-dioxin		*F. pulverulenta*	[24]
(**199**) 2-formyl-5-(hydroxymethyl)furan		*F. hirsuta*	[32]
(**200**) 2-methyldecanal		*F. laevis*	[43]
(**201**) (*Z*)-12-hydroxy-14-methyl-oxacyclotetradec-6-en-2-one		*F. pulverulenta*	[24]
(**202**) 7-(1-hydroxypentyl)-2-oxabicyclo[3.3.0]oct-7-en-3-one	R = C_4_H_9_	“	“
(**203**) citric acid		*F. laevis*	[13]
(**204**) 12-oxophytodienoic acid		“	“
(**205**) tuberonic acid sulfate		“	“
(**206**) phthalic acid, butyl tetradecyl ester	R1 = *n*Bu; R2 = tetradecyl	*F. hirsuta*	[32]
(**207**) phthalic acid, isobutyl octadecyl ester	R1 = *i*Bu; R2 = octadecyl	“	“
(**208**) *N,N’*-bis(carbobenzyloxy)-l-lysinyl-l-valine methyl ester		“	“
(**209**) 5-phenyloxymethyl-2-phenylhydrazino-4,5-dihydro-1,3-oxazole		“	“
(**210**) 2,5-dihydroperoxy-2,5-dimethylhexane		“	“

^a^ The mark “ indicates that the same species as above is concerned. ^b^ The mark “ indicates that the same reference as above is concerned.

## Data Availability

The original contributions presented in the present study are included in the cited articles; further inquiries can be directed to the corresponding authors.
